# Oncologic relevance of genetic alterations in sporadic synchronous and solitary colorectal cancer: a retrospective multicenter study

**DOI:** 10.1186/s12876-023-02937-7

**Published:** 2023-09-04

**Authors:** Il Tae Son, Minsung Kim, Bo Young Oh, Min Jeong Kim, Sang Nam Yoon, Jun Ho Park, Byung Chun Kim, Jong Wan Kim

**Affiliations:** 1 Department of Surgery, Hallym Sacred Heart Hospital, Hallym University College of Medicine, Anyang Si, Republic of Korea; 2 Department of Surgery, Kangdong Sacred Heart Hospital, Hallym University College of Medicine, Seoul, Republic of Korea; 3 Department of Surgery, Kangnam Sacred Heart Hospital, Hallym University College of Medicine, Seoul, Republic of Korea; 4 Department of Surgery, Dongtan Sacred Heart Hospital, Hallym University College of Medicine, 40, Sukwoo-Dong, Hwaseong-Si, Gyeonggi-Do, Republic of Korea

**Keywords:** Synchronous colorectal cancer, Gene mutation, Survival

## Abstract

**Background:**

Oncologic impact of genetic alteration across synchronous colorectal cancer (CRC) still remains unclear. This study aimed to compare the oncologic relevance according to genetic alteration between synchronous and solitary CRC with performing systematic review.

**Methods:**

Multicenter retrospective analysis was performed for CRC patients with curative resection. Genetic profiling was consisted of microsatellite instability (MSI) testing, *RAS* (K-ras, and N-ras), and *BRAF* (v-Raf murine sarcoma viral oncogene homolog B1) V600E mutation. Multivariate analyses were conducted using logistic regression for synchronicity, and Cox proportional hazard model with stage-adjusting for overall survival (OS) and disease-free survival (DFS).

**Results:**

It was identified synchronous (*n* = 36) and solitary (*n* = 579) CRC with similar base line characteristics. *RAS* mutation was associated to synchronous CRC with no relations of MSI and *BRAF*. During median follow up of 77.8 month, Kaplan–meier curves showed significant differences according to MSI-high for OS, and in *RAS*, and *BRAF mutation* for DFS, respectively. In multivariable analyses, *RAS* and *BRAF* mutation were independent factors (*RAS*, HR = 1.808, 95% CI = 1.18–2.77,* p* = 0.007; *BRAF*, HR = 2.417, 95% CI = 1.32–4.41, *p* = 0.004). Old age was independent factor for OS (HR = 3.626, 95% CI = 1.09–12.00, *p* = 0.035).

**Conclusion:**

This study showed that oncologic outcomes might differ according to mutation burden characterized by *RAS, BRAF*, and MSI between synchronous CRC and solitary CRC. In addition, our systematic review highlighted a lack of data and much heterogeneity in genetic characteristics and survival outcomes of synchronous CRC relative to that of solitary CRC.

## Introduction

Multiplicity in colorectal cancer (CRC) is defined as a synchronous cancer in which at least one additional colorectal tumor is detected simultaneously to the initially diagnosed primary CRC in a single individual. Although synchronous CRC accounts for a small proportion of cases, approximately 2%–10% of all CRCs [1, 2], it differs from solitary CRC in terms of the extent of curative resection required for the tumor location [3] and molecular genomic heterogeneity [4, 5] because tumor carcinogenesis is influenced by genetic, epigenetic, and environmental factors [6]. Those complexities can affect the management and prognosis of synchronous CRC.

Previous studies have compared the clinicopathologic features and prognosis between synchronous and solitary CRC [3, 7–14]. However, some studies included CRC patients with a metachronous tumor or stage IV cancer and others lacked data for synchronous CRC-related gene information or survival outcomes in large populations. In our prior literature reviews [15, 16], we identified heterogeneity in the oncologic outcomes in studies comparing both cancers [1, 6, 9, 11–14, 17, 18]. Study populations that include patients with Lynch syndrome or familial adenomatous polyposis might exhibit selection bias, as synchronous CRCs harboring multiple genes associated with hereditary tumors rather than sporadic tumors can show heterogeneous prognosis [6, 16, 19–21]. Synchronous CRCs are frequently characterized by microsatellite instability (MSI), which is caused by epigenetic inactivation of the *MLH1* gene via promoter methylation, whereas Lynch syndrome is caused by germline mutations in the mismatch repair (MMR) genes [22]. Furthermore, the V600E mutation in *BRAF* (v-Raf murine sarcoma viral oncogene homolog B1) in MSI-high CRC with methylation of *MLH1* promoters is associated with poor prognosis and decreased likelihood of Lynch syndrome [23]. Previous studies have established the prognostic roles of MSI status, *BRAF* mutations, and *RAS* mutations in solitary CRC with concordance for MSI-deficient status [23–26]. However, the oncologic impact of these genetic factors in synchronous CRC, relative to solitary CRC, remains unclear [27].

Therefore, the aims of this study were to compare the oncologic relevance of *BRAF* mutations, *KRAS* mutations, and MSI status between synchronous and solitary CRC. In addition, we performed a systematic review of prior studies that compared the genetic status and survival outcomes between synchronous and solitary CRC.

## Methods

We retrospectively reviewed the medical records of CRC patients who underwent curative surgery at four tertiary hospitals (Hallym Sacred Heart Hospital, Dontan Sacred Heart Hospital, Kangnam Sacred Heart Hospital, and Kangdong Sacred Heart Hospital) between March 2014 and December 2020. Patients diagnosed with familial adenomatous polyposis, hereditary non-polyposis colorectal cancer (HNPCC), and patients with inflammatory bowel disease combined with a metachronous malignancy were excluded from this study. Patients diagnosed with clinical or pathological stage IV CRC and patients without genetic information regarding their MSI status, *BRAF* mutations, and *RAS* mutations were also excluded. The study protocol was approved by the institutional review board (a central IRB No. 2022–12-022). The review board waived the requirement for informed consent because this study involved retrospective analyses.

Synchronous CRC was defined as follows: each lesion must be diagnosed as malignant, separate entities and must not be metastases of another tumor; and the synchronous lesions must be diagnosed simultaneously or within 6 months of diagnosis of the first tumor. For synchronous cancer, the most pathologically advanced lesion was defined as the index tumor. The tumor location and pathological status were defined relative to the index tumor. Asymptomatic patients were diagnosed in healthcare screening. Surgical resection was classified into three categories. Single segmental resection was defined as radical resection. Multiple segmental resections were defined as two radical resections with two anastomoses. Extended resection included total colectomy, subtotal collection, or total proctocolectomy. The surgical procedure, use of adjuvant/neoadjuvant therapy, and postoperative surveillance were determined by the attending physician based on the pathologic stage and the general condition of the patient in accordance with the National Comprehensive Cancer Network guideline [28].

After histologic examination, we performed real-time polymerase chain reaction analysis of *BRAF* codon 600 and MSI status, and peptide nucleic acid clamp of *KRAS* (mutation in codons 12 and 13 of exon 2, codon 61 of exon 3, and codon 146 of exon 4), and *NRAS* (mutation of exons 2, 3, and 4) [29–32]. *RAS* mutations were defined as mutations in *KRAS* or *NRAS*. MSI status was determined using the markers BAT25, BAT26, NR21, NR24, and NR27 and classified as MSI-high (two or more unstable markers) or microsatellite stable (MSS; one or no unstable marker).

Categorical variables were compared using Pearson’s χ^2^ test, and continuous variables were compared using Student’s *t* test. Multivariable binomial logistic regression analysis was performed to evaluate associations between the genetic factors and CRC synchronicity. The Kaplan–Meier method with the log-rank test was used to assess disease-free survival (DFS) and overall survival (OS) in patients stratified by *RAS* mutations*, BRAS* mutations, and MSI status. The proportional hazard ratio model with adjustment for pathological stage was used for multivariable analyses of DFS and OS. In all analyses, *p*-values of < 0.05 were considered statistically significant. All statistical analyses were performed using SPSS version 25.0 (SPSS Inc., Chicago, IL, USA).

We also performed systematic searches of PubMed, Cochrane Library, EMBASE, Medline, and Web of Science to identify all of the available studies on synchronous CRC that had been published and indexed up to October 31, 2022. We searched for comparative studies of solitary and synchronous or multiple CRC and excluded studies of metachronous CRC and noncomparative studies. Medical Subject Headings (MeSH) terms and Emtree terms were used in PubMed and EMBASE, respectively, together with separate words or word combinations to search the title or abstract.

## Results

Of 615 patients available in the medical records, 36 patients (5.9%) were diagnosed for with synchronous CRC. The clinical characteristics were similar between the solitary CRC and synchronous CRC patients (Table 1). Multiple segmental resection or extended resection was performed in a greater proportion of synchronous CRC patients than solitary CRC patients (*p* < 0.001). Lymph node metastasis was more common (*p* = 0.037), and the number of harvested and retrieved lymph nodes was significantly greater (*p* = 0.002) in synchronous CRC patients (Table 2). Regarding genetic mutations, *RAS* mutations were more frequent in solitary CRC patients than in synchronous CRC patients (51.6% *vs.* 16.7, *p* < 0.001; Table 3). A multivariable analysis showed that the presence of *RAS* mutations was significantly associated with reduced risk of synchronous CRC, with an odds ratio (OR) of 0.184 (95% confidence interval [CI] 0.07–0.45, *p* = 0.001). In addition, when we assessed genetic mutations stratified by tumor location, *RAS* mutations were more frequently detected in solitary CRC for both right-sided (56.6% *vs*. 6.3%, *p* < 0.001) and left-sided (52.2% *vs*. 21.4%, *p* = 0.026) tumors, whereas no differences were found for MSI status or *BRAF* mutations. The multivariable analyses showed that synchronous CRCs were less frequently associated with *RAS* mutations for both right-sided (OR = 0.05, 95% CI 0.01–0.39, *p* = 0.004) and left-sided (OR = 0.025, 95% CI 0.07–0.92, *p* = 0.038) tumors. MSI status and *BRAF* mutations were not associated with tumor location (Table 4).

**Table 1:** Clinical characteristics of the patients between solitary and synchronous CRC patients

Variables	Solitary CRC (*n* = 579)	Synchronous CRC (*n* = 36)	*p* value
Age, mean ± SD	67.1 ± 13.0	65.9 ± 12.0	0.605
≥ 60	416 (71.8)	25 (69.4)	0.756
< 60	163 (28.2)	11 (30.6)	
Gender, n (%)			0.554
Male	238 (41.1)	13 (36.1)	
Female	341 (58.9)	23 (63.9)	
BMI (kg/m^2^), mean ± SD	23.7 ± 3.6	24.7 ± 4.8	0.247
ASA score, n (%)			0.159
I-II	333 (57.5)	25 (69.4)	
III-V	246 (42.5)	11 (30.6)	
Symptoms, n (%)			0.293
Yes	341 (58.9)	18 (50.0)	
No	238 (41.1)	18 (50.0)	
Clinical perforation, n (%)			0.775
Yes	12 (2.1)	1 (2.8)	
No	567 (97.9)	35 (97.2)	
Clinical obstruction, n (%)			0.692
Yes	112 (19.3)	6 (16.7)	
No	467 (80.7)	30 (83.3)	
CEA, n (%)			0.795
≥ 6.0	189 (32.6)	11 (30.6)	
< 6.0	390 (67.4)	25 (69.4)	
Tumor location, n (%)			0.077
Right-sided	196 (33.8)	16 (44.4)	
Left-sided	180 (31.1)	14 (38.9)	
Rectum	203 (35.1)	6 (16.7)	
Approach, n (%)			0.430
Open	47 (8.1)	4 (11.1)	
Conventional laparoscopy	396 (68.4)	28 (77.7)	
Single port laparoscopy	16 (2.7)	0 (0)	
Robot-assisted	89 (15.4)	2 (5.6)	
Open conversion	31 (5.4)	2 (5.6)	
Operation type, n (%)			< 0.001
Single segmental resection	573 (99.0)	26 (72.2)	
Multiple segmental resection	0 (0)	7 (19.5)	
Extended resection	6 (1.0)	3 (8.3)	
Adjuvant chemotherapy, n (%)			0.058
Yes	260 (44.9)	22 (61.1)	
No	319 (55.1)	14 (38.9)	

*CRC* Colorectal cancer, *SD* Standard deviation, *BMI* Body mass index, *ASA* American society of anesthesiologist, *CEA* Carcinoembryonic antigen

**Table 2:** Pathologic features of the patients between solitary and synchronous CRC patients

Variables	Solitary CRC (*n* = 579)	Synchronous CRC (*n* = 36)	*p* value
Differentiation, n (%)			0.077
Well differentiated	110 (19.0)	2 (5.6)	
Moderate differentiated	439 (75.8)	31 (86.1)	
Poorly differentiated	21 (3.6)	3 (8.3)	
Mucinous/signet ring cell	9 (1.6)	0 (0)	
Stage, n (%)			0.241
0	21 (3.6)	1 (2.8)	
I	135 (23.3)	5 (13.8)	
II	214 (37.0)	11 (30.6)	
III	209 (36.1)	19 (52.8)	
T stage, n (%)			0.436
0/Tis	23 (4.0)	1 (2.8)	
1	80 (13.8)	5 (13.9)	
2	72 (12.4)	1 (2.8)	
3	331 (57.2)	25 (69.4)	
4	73 (12.6)	4 (11.1)	
N stage, n (%)			0.037
0	370 (63.9)	17 (47.2)	
1	130 (22.5)	15 (41.7)	
2	79 (13.6)	4 (11.1)	
Number of retrieved LN, mean ± SD	23.1 ± 13.3	30.1 ± 15.0	0.002
LVI, n (%)			0.151
Positive	220 (38.0)	18 (50.0)	
Negative	359 (62.0)	18 (50.0)	
PNI, n (%)			0.696
Positive	144 (24.9)	10 (27.8)	
Negative	435 (75.1)	26 (72.2)	

*CRC* Colorectal cancer, *LN* Lymph node, *SD* Standard deviation, *LVI* Lymphovascular invasion, *PNI* Perineural invasion, *MSI* Microsatellite instability, *MSI-H* Microsatellite instability high, *MSS* Microsatellite stable

**Table 3:** Distribution of gene mutation and risk for synchronicity of colorectal cancer

Genetic profile	χ^2^ analysis			Univariate^b^		Multivariate^b^	
	Solitary CRC (*n* = 579)	Synchronous CRC (*n* = 36)	*p*	OR (95% CI)	*p*	OR (95% CI)	*p*
MSI			0.723		0.648		0.916
MSS	542 (93.6)	33 (91.7)		Reference		Reference	
MSI-H	37 (6.4)	3 (8.3)		0.751 (0.22–2.56)		0.932 (0.25–3.43)	
*RAS* mutation			0.001		0.001		0.001
No	280 (48.4)	30 (83.3)		Reference		Reference	
Yes	299 (51.6)	6 (16.7)		0.187 (0.07–0.45)		0.184 (0.07–0.45)	
*BRAF* mutation^a^			1.0		0.860		0.934
No	347 (59.9)	22 (61.1)		Reference		Reference	
Yes	63 (10.9)	4 (11.1)		1.071 (0.49–2.31)		0.967 (0.44–0.2.11)	

*CRC* Colorectal cancer

^a^Not available data

^b^binomial logistic regression model for synchronicity of CRC

**Table 4:** Genetic profiling for solitary and synchronous colorectal cancer according to tumor location

Variables	Right-sided					Left-sided					Rectum				
	χ^2^ analysis			Multivariate analysis		χ^2^ analysis			Multivariate analysis		χ^2^ analysis			Multivariate analysis	
	Solitary CRC (*n* = 196)	Synchronous CRC (*n* = 16)	*p*	HR (95% CI)	*p*	Solitary CRC (*n* = 180)	Synchronous CRC (*n* = 14)	*p*	HR (95% CI)	*p*	Solitary CRC (*n* = 203)	Synchronous CRC (*n *= 6)	*p*	HR (95% CI)	*p*
MSI			0.726					1					1		
MSS	165 (84.2)	13 (81.2)		Reference		176 (97.8)	14 (100)		Reference		201 (99.0)	6 (100)		Reference	
MSI-H	31 (15.8)	3 (18.8)		0.60 (0.15–2.43)	0.472	4 (2.2)	0 (0)		0 (0)	0.999	2 (1.0)	0 (0)		0 (0)	0.999
RAS mutation			< 0.001					0.026					0.689		
No	85 (43.4)	15 (93.7)		Reference		86 (47.8)	11 (78.6)		Reference		109 (53.7)	4 (66.7)		Reference	
Yes	111 (56.6)	1 (6.3)		0.05 (0.01–0.39)	0.004	94 (52.2)	3 (21.4)		0.25 (0.07–0.92)	0.038	94 (46.3)	2 (33.3)		0.61 (0.11–3.44)	0.578
BRAF mutation			1					0.449					0.561		
No	106 (54.1)	9 (56.2)		Reference		124 (68.9)	8 (57.1)		Reference		117 (57.6)	5 (83.3)		Reference	
Yes	36 (18.4)	3 (18.8)		0.68 (0.16–2.92)	0.6	17 (9.4)	1 (7.1)		0.87 (0.10–7.53)	0.897	10 (4.9)	0 (0)		0 (0)	0.999
Unknown	54 (27.6)	4 (25.0)		0.98 (0.27–3.57)	0.979	39 (21.7)	5 (35.7)		2.65 (0.78–8.99)	0.119	76 (37.4)	1 (16.7)		0.31 (0.04–2.72)	0.291

*HR* Hazard ratio, *CI* Confidence interval, *MSI* Microsatellite instability, *MSS* Microsatellite stable, *MSI-H* Microsatellite instability high

**Fig. 1:**
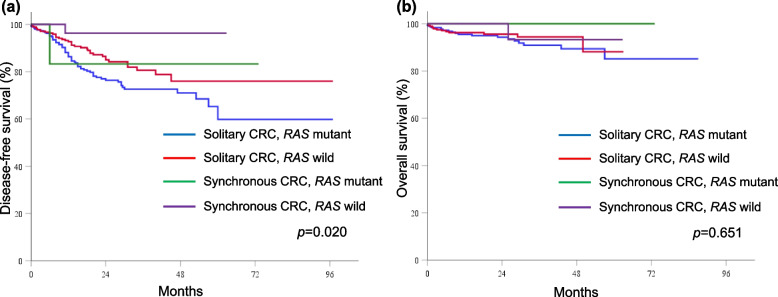
The Kaplan–Meier curves stratified by *RAS* mutation status and tumor type. (**a**) disease-free survival and (**b**) overall survival

**Fig. 2:**
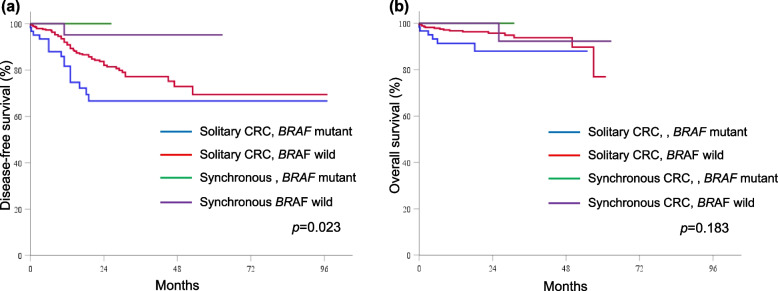
The Kaplan–Meier curves stratified by *BRAF* mutation status and tumor type. (**a**) disease-free survival and (**b**) overall survival

**Fig. 3:**
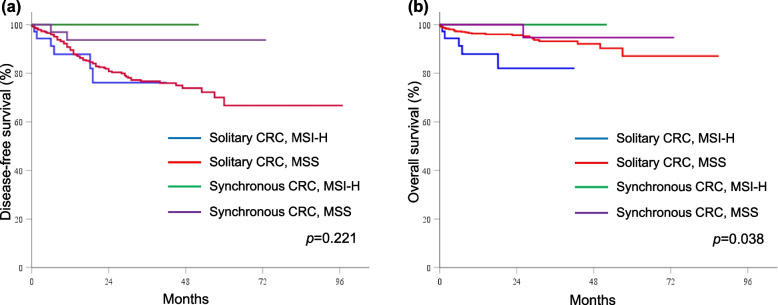
The Kaplan–Meier curves stratified by microsatellite instability status and tumor type. (**a**) disease-free survival and (**b**) overall survival

The median follow-up duration was 22.2 (range 1.0–89.1) months in patients with solitary CRC and 27.9 (range 10.1–73.3) months in patients with synchronous CRC. The DFS and OS rates were similar between solitary and synchronous CRC patients (DFS: 94.4% vs 82.6%, *p* = 0.041; OS: 94.3% vs 97.2%, *p* = 0.333). The Kaplan–Meier curves of DFS stratified by mutation status and tumor type revealed that DFS was worst for solitary CRC patients with *RAS* mutations (*p* = 0.020; Fig. 1a) or *BRAF* mutations (*p* = 0.023; Fig. 2a). However, there were no significant differences in OS according to the *RAS* (*p* = 0.651; Fig. 1b) or *BRAF* (*p* = 0.183; Fig. 2b) mutation status. Solitary CRC patients with MSI-high had the worst OS (*p* = 0.038; Fig. 3b), but not in DFS (*p* = 0.221; Fig. 3a). In the univariate analyses, pathologic stage, presence of *RAS* mutations*,* and presence of *BRAF* mutations were risk factors for DFS, whereas old age and MIS-high status were risk factors for OS. In the stage-adjusted multivariable analyses, *RAS* (hazard ratio [HR] 1.808, 95% CI 1.18–2.77,* p* = 0.007) and *BRAF* (HR 2.417, 95% CI 1.32–4.41, *p* = 0.004) mutations were independent risk factors for DFS, and old age was the only independent risk factor for OS (HR 3.626, 95% CI = 1.09–12.00, *p* = 0.035; Table 5).

**Table 5:** Univariate and multivariate analyses for disease-free survival and overall survival

Variables	Disease-free survival				Overall survival			
	Univariate		Multivariate^a^		Univariate		Multivariate^a^	
	HR (95% CI)	*p*	HR (95% CI)	*p*	HR (95% CI)	*p*	HR (95% CI)	*p*
Age								
< 60	Reference		Reference		Reference		Reference	
≥ 60	1.27 (0.81–1.98)	0.293	1.076 (0.68–1.68)	0.750	4.44 (1.36–14.53)	0.014	3.626 (1.09–12.00)	0.035
Gender								
Male	Reference				Reference			
Female	1.29 (0.88–1.91)	0.197			0.95 (0.48–1.90)	0.89		
Tumor location								
Right-sided	Reference				Reference			
Left-sided	0.82 (0.51–1.32)	0.412			0.78 (0.34–1.82)	0.572		
Rectum	0.89 (0.56–1.42)	0.634			0.89 (0.40–1.99)	0.772		
Stage								
0-II	Reference				Reference			
III	1.93 (1.31–2.85)	0.001			1.73 (0.88–3.39)	0.11		
MSI								
MSS	Reference		Reference		Reference		Reference	
MSI-H	1.16 (0.51–2.65)	0.728	1.061 (0.43–2.58)	0.896	3.09 (1.19–8.05)	0.021	2.59 (0.87–7.70)	0.085
*RAS* mutation								
No	Reference		Reference		Reference		Reference	
Yes	1.72 (1.15–2.57)	0.008	1.808 (1.18–2.77)	0.007	1.34 (0.67–2.65)	0.408	1.443 (0.67–3.07)	0.341
*BRAF* mutation								
No	Reference		Reference		Reference		Reference	
Yes	1.92 (1.10–3.35)	0.021	2.417 (1.32–4.41)	0.004	2.38 (0.93–6.05)	0.07	1.97 (0.71–5.47)	0.191
Synchronicity								
No	Reference		Reference		Reference		Reference	
Yes	0.259 (0.06–1.05)	0.059	0.318 (0.7–1.30)	0.112	0.388 (0.05–2.83)	0.351	0.313 (0.04–2.39)	0.313

*HR* Hazard ratio, *CI* Confidence interval, *MSI* Microsatellite instability, *MSI-H* Microsatellite instability high, *MSS* Microsatellite stable

^a^Pathologic stage-adjusted regression model

## Discussion

This study showed that DFS might be influenced by the mutation burden, independent to clinical factors, or tumor burden in synchronous and solitary CRC. The genetic profiles revealed that *RAS* and *BRAF* mutations were associated with more pronounced effects than MSI on DFS. In this study, old age was the only risk factor for OS, regardless of the mutation profile and pathologic stage, and OS was not associated with MSI status in synchronous CRC. By contrast, a previous study suggested that, due to the concordance between MSI status and synchronicity, older individuals are more likely to develop multiple cancers through the MSI pathway, secondary to a widespread CpG island methylator phenotype (CIMP) and silencing of the MMR gene *MLH1* by genetic and or environmental factors [6, 33]. However, the systematic review of studies comparing synchronous and solitary CRC revealed a lack of published data, as well as much heterogeneity in genetic and survival information, with unclear associations of clinical factors and genetic profiles with the prognosis of synchronous CRC (Table 6). Thus, we consider that the association between genetic mutations and the prognosis of synchronous CRC patients remains unclear and is open to debate. Undetermined association might implicate a complex hypothetical predisposition of development to synchronous CRC. Field effect as cancerization concept of molecular alterations induced by global DNA methylation such as LINE-1 methylation, MGMT promoter methylation or other CpG island methylation, has been proposed to explain the development of multiple primary malignancies in the same organ [34]. The epigenetic field effect can drive concordant or discordant genetic patterns in synchronous cancer pairs, which also exhibit different phenotypes depending on the MSI status, as a confounding effect that can lead to a worse prognosis than the corresponding solitary tumor [33]. Therefore, we suggest that many contributing factors to the controversy over the prognosis for synchronous CRC patients should be addressed in a controlled dataset of clinical, pathological, genetic, and survival information, accompanied by intensive surveillance.

**Table 6:** A systematic review of synchronous CRC compared with solitary CRC

Study	Design	Age	Gender		Tumor location			MSI		Ras mutation		BRAF mutation		Oncologic outcomes	Finding
			Male	Female	Right	Left	Rectum	MSI-H	MSS	Yes	No	Yes	No		
Bae JM et al. 2012 [1]	RCS														More common synch CRC in male and colon More frequent MSI-H in synch CRC
Synch	46 (30)	60	34 (74)*	12 (26)*	Total tumor 31 (32)	Total tumor 43 (44)	Total tumor 24 (24)	Total tumor 27 (28)*	Total tumor 71 (72)*	Total tumor 23 (24)	Total tumor 75 (76)	Total tumor 4 (4)	Total tumor 94 (96)	Mean OS 68 months Mean PFS 59 months	
Solitary	105 (70)	62	59 (56)*	46 (44)*	30 (29)	49 (46)	26 (25)	6 (6)*	99 (94)*	26 (25)	79 (75)	4 (4)	101 (96)	Mean OS 55 months Mean PFS 47 months	
Lee BC et al. 2017 [3]	RCS														More common synch CRC in male, older patients, and left-sided
Synch	217 (3)	62.3*	156 (72)*	61 (28)*	152 (33)*	192 (42)*	115 (25)*	23 (13)*	194 (87)*						
Solitary	7984 (97)	59.8*	4886 (61)*	3098 (39)*	1739 (22)*	2711 (34)*	3511 (44)*	342 (7)*	7642 (93)*						
Nosho K et al. 2009 [6]	PCS														Synch CRC had more frequent mutations in BRAF, and MSI, and had a worse prognosis than solitary CRC
Synch	47 (2)	65.6*	17 (36)	30 (64)	23 (51)	16 (36)	6 (13)	7 (30)*	16 (70)*	8 (35)	15 (65)	8 (35)*	15 (65)*	5-year CSS 62%* 5-year OS 61%*	
Solitary	2021 (98)	68.9*	612 (30)	1409 (70)	845 (43)	680 (35)	447 (23)	118 (14)*	722 (86)*	314 (37)	537 (63)	97 (12)*	731 (88)*	5-year CSS 74%* 5-year OS 70%*	
Dykes SL et al. 2003 [7]	RCS				MSS 28 (24)* MSI-H 44 (81)*	MSS 88 (76)* MSI -H 10 (19)*		116 (68)	54 (32)						Concordance in MSI/MSS status among tumors in the same individual
Synch	77 (3)	73*	(56)	(44)						NA	NA	NA	NA	NA	
Solitary	2884 (97)	68*	(52)	(48)						NA	NA	NA	NA	NA	
Lam AK et al. 2011 [8]	RCS														More frequent synch CRC in males and the right colon
Synch	102 (5)	68	69 (68)*	33 (32)*	100 (44)*	79 (35)*	47 (21)*	NA	NA	NA	NA	NA	NA	5-year CSS 53%	
Solitary	1793 (95)	67	959 (53)*	834 (47)*	612 (34)*	581 (32)*	600 (34)*	NA	NA	NA	NA	NA	NA	5-year CSS 53%	
Mulder SA et al. 2011 [9]	RPS														More frequent synch CRC in males and patients aged over 70 years More frequent synch CRC in the colon than the rectum
Synch	534 (4)	< 70 196 (37)* ≥ 70 338 (63)*	323 (61)*	211 (39)*	193 (36)*	261 (49)*	80 (15)*	NA	NA	NA	NA	NA	NA	5-year OS 58%*	
Solitary	13,683 (96)	< 70 6084 (46)* ≥ 70 7065 (54)*	6723 (51)*	6423 (49)*	4337 (33)*	5724 (44)*	3088 (23)*	NA	NA	NA	NA	NA	NA	5-year OS 64%*	
van Leersum NJ et al. 2014 [10]	RPS														Synch CRC associated with a higher risk of severe postoperative complications and reinterventions
Synch	884 (4)	72.2*	537 (61)*	347 (39)*	744 (43)*	701 (38)*	323 (19)*	NA	NA	NA	NA	NA	NA	NA	
Solitary	24,529 (96)	69.7*	13,319 (55)*	11,210 (45)*	9095 (37)*	8421 (34)*	7013 (29)*	NA	NA	NA	NA	NA	NA	NA	
Kato T et al. 2016 [11]	RCS														Advanced age and left colon tumor location associated with higher risk of synch CRC
Synch	84 (8)	70.3*	62 (74)*	22 (26)*	26 (31)*	37 (44)*	21 (25)*	NA	NA	NA	NA	NA	NA	5-year OS 74.5%	
Solitary	921 (92)	67.1*	575 (62)*	346 (38)*	308 (33)*	282 (31)*	331 (36)*	NA	NA	NA	NA	NA	NA	5-year OS 75.7%	
Arakawa K et al. 2018 [12]	RCS														Synch CRC had a poorer RFS than solitary CRC
Synch	92 (7)	69	54 (59)	38 (41)	21 (23)	71 (77)		NA	NA	NA	NA	NA	NA	5-year RFS 65.3%*	
Solitary	1203 (93)	67	699 (58)	504 (42)	377 (31)	826 (69)		NA	NA	NA	NA	NA	NA	5-year RFS 75.1%*	
Latournerie M et al. 2008 [13]	RPS														More frequent synch CRC in males and patients aged over 65 years No survival difference between synch CRC and solitary CRC
Synch	596 (4)	< 65 138 (23)* ≥ 65 458 (77)*	387 (65)*	209 (35)*	183 (31)	295 (49)	118 (20)	NA	NA	NA	NA	NA	NA	5-year OS 38.9%	
Solitary	14,966 (96)	< 65 4111 (27)* ≥ 65 10,842 (73)*	8216 (55)*	6750 (45)*	NA	NA	NA	NA	NA	NA	NA	NA	NA	5-year OS 38.6%	
Derwinger K et al. 2011 [14]	RCS														Older Synch CRC patients than solitary CRC Female with synch CRC had a better survival outcome than male with synch CRC
Synch	60 (2)	72.9*	35 (58)	25 (42)	Colon 37 (62)		Rectum 23 (38)	NA	NA	NA	NA	NA	NA	CSS Female > male OS Female > male	
Solitary	2464 (98)	69.2*	1220 (50)	1244 (50)	Colon 1531 (62)		Rectum 933 (38)	NA	NA	NA	NA	NA	NA	NA	
Hu H et al. 2013 [17]	RCS														Synch CRC had more frequent Synch CRC patients in MSI-H tumors and better OS compared with solitary CRC
Synch	58 (35)	70*	30 (52)	28 (48)	Right only 23 (40)*	Left/ rectum only 16 (28)*	Right and left/ rectum 19 (33)*	21 (36)*	37 (64)*					5-year OS 92%*	
Solitary	109 (65)	60*	67 (61)	42 (39)	Right only 47 (43)*	Left/ rectum only 62 (57)*	Right and left/ rectum 0 (0)*	13 (12)*	96 (88)*					5-year OS 56%*	
Warps AK et al. 2021 [18]	RPS														Most synch CRC located on the right side Bilateral synch CRC resulted in a higher postoperative complication and mortality
Synch	3095 (3)	Colon < 60 227 (11)* ≥ 60 1919 (89)* Rectum < 60 132 (14)* ≥ 60 817 (86)*	Colon 1162 (54) Rectum 694 (73)*	Colon 984 (46) Rectum 255 (27)*	Right-right colon 903 (42)*	Left-left colon 544 (25)* Right-left colon 699 (33)*	Rectum-rectum 165 (17)* Rectum-right colon 352 (34)* Rectum-left colon 459 (48)*	NA	NA	NA	NA	NA	NA	NA	
Solitary	97,397 (97)	Colon < 60 11,676 (17)* ≥ 60 66,150 (83)* Rectum < 60 7203 (25)* ≥ 60 21,338 (75)*	Colon 36,122 (52) Rectum 17,929 (63)*	Colon 32,697 (48) Rectum 10,607 (37)*	Right colon 36,244 (53)*	Left colon 32,609 (47)*	Rectum 28,546 (100)*	NA	NA	NA	NA	NA	NA	NA	
Fukatsu H et al. 2007 [35]															Varied concurrent adenomas of synchronous cancer according to tumor location
Synch	249 (8)	67.3	175 (70)*	74 (30)*	39 (16)	122 (49)	Right and Left 88 (35)	NA	NA	NA	NA	NA	NA	NA	
Solitary	2812 (92)	66.4	1624 (58)*	1188 (42)*	NA	NA	NA	NA	NA	NA	NA	NA	NA	NA	
He W et al. 2019 [36]	RCS PSM														Synch CRC has a poor survival outcome compared with solitary CRC
Synch	126 (50)	< 60 47 (37) ≥ 60 79 (63)	84 (67)	84 (67)	Colon 67 (53)		Rectum 59 (47)							5-year OS 65.7%* 5-year DFS 55.4%* 5-year CSS 67.7%*	
Solitary	126 (50)	< 60 49 (39) ≥ 60 77 (61)	42 (33)	42 (33)	Colon 67 (53)		Rectum 59 (47)							5-year OS 81.6%* 5-year DFS 75.7%* 5-year CSS 83.5%*	

*CRC* Colorectal cancer, *Synch* Synchronous, *PCS* Prospective cohort study, *RCS* Retrospective cohort study, *RPS* Retrospective population-based study, *PSM* Propensity score-matched analysis;

Study including patients with hereditary colorectal cancer, or Lynch syndrome or, inflammatory bowel disease or, familial adenomatous polyposis or, metachronous cancer

^*^valuse with a statistical significance

As a commonly identified genotype in multiple CRCs, MSI arising from promoter methylation of the biallelic *hMLH1* gene differs to that arising from the HNPCC pathway [37]. In sporadic CRC, the risk of synchronicity was higher (2.14-fold) in patients with MSI than in patients with MSS, but there were no relationships between clinical features and the MSI genotype [38]. Global hypermethylation of colorectal epithelium, as independent events, could increase the frequency of multiple CRCs in older age instead of developing from a predisposition to cancer in patients with sporadic MSI CRC [20]. In terms of the oncologic outcomes, the association between MSI status and greater tumor burden in synchronous CRC suggests that synchronicity and *BRAF* mutations are risk factors for OS in patients with MSS CRC, but the disease-specific survival of MSI CRC patients was unaffected by synchronicity in a stage-adjusted analysis [33]. By contrast, a prospective cohort study found that the overall mortality was greater in synchronous CRC patients than in solitary CRC patients, and the authors reported that multiple colon cancers arose through the serrated pathway, which is characterized by high frequencies of *BRAF* mutations, CIMP-high, and MSI-high [6]. Another study reported contradictory results regarding the rarity of *BRAF* c.1799T4A mutation in synchronous advanced malignancies as a stage-independent predictor of poor prognosis in association with MSS, which is incompatible with the various epigenetic defects of synchronous CRCs [33].

Poor oncologic outcomes of synchronous CRC, in terms of the OS, DFS, and cancer-specific survival (CSS), were reported in a previous study using a matched-pairs analysis [36] (Table 6). Following disease relapse, it is important to select the most appropriate molecular-targeted drug by performing biomarker analysis [12]. However, the greater and heterogenous mutation burden of paired tumors makes it difficult to identify the most appropriate target, resulting in poor prognosis of patients with relapsed synchronous CRC. Adjuvant therapeutic strategies have not yet been established for relapsed synchronous CRC, and we are still dependent on the clinical guidelines for solitary CRC. For synchronous CRCs within the same patient, it has been reported that paired lesions display heterogeneity in canonical genes, including *APC*, *KRAS*, *TP53*, and *PIK3CA*, together with a high frequency of mutations, compared with solitary CRC. Therefore, when drugs such as vemurafenib and dabrafenib, which target the *BRAF* mutation pV600E, are used to treat one lesion, the other lesion might be unresponsive due to the heterogeneous mutation profile of paired synchronous CRCs [39]. Those molecular profiles develop independently, and lesions present with different gene copy numbers resulting in unique gene signatures in each lesion, combined with clonal mutations at different loci and accumulated timing [4]. When treating patients with *BRAF*-mutated synchronous CRC, in particular, the MSI status and genetic heterogenicity of the paired tumors should be considered rather than the tumor burden or clinical stage. According to a systematic review of patients with *BRAF*-mutated CRC, MSS was associated with worse prognosis than MSI, but the clinical stratification by MSI testing and heterogeneity of genetic mutations have not been established for patients with *BRAF*-mutated synchronous CRC [40]. Physician should also be aware that the poor prognosis of synchronous cancers might be independent of genetic factors such as *BRAF* mutations, MSI-high, and CIMP-high due to unidentified molecular events caused by the genetic or environmental background [6]. Several markers, such as the transcriptional effector *RPL22,* a candidate gene involved in nodal/transforming growth factor-β and the ribosomal protein–murine double minute 2 (MDM2)–p53 signaling pathway [4], as well as different methylation rates of *CACNA1G*, *NEUROG1*, and *CDKN2A* (p16) [1], might confound analyses of the prognosis of synchronous CRC.

Some studies have also demonstrated similar or better prognosis of synchronous CRC patients compared with solitary CRC patients, regardless of CIMP status and *KRAS* or *BRAF* mutations [1]. This prognostic pattern was observed in several studies that lacked genetic information [8, 9, 11, 13] (Table 6). Although there was no clear explanation for this finding, intensive perioperative colonoscopy detected associated adenomas that are more prone to progress into multiple colorectal cancers in old patients and slow growing tumors with an uncharacterized predisposition [11, 13, 35]. In addition, advanced surgical procedures could achieve comparable long-term outcomes for synchronous CRC patients, regardless of whether they underwent resection of more than two regions or extensive resection of a single region [3].

This study has some limitations, including a small sample size due to the exclusion of synchronous CRC patients without genetic information, which reduced the statistical power. The absence of data regarding CIMP status and germline mutation of MMR genes might also introduce bias in terms of assessing whether the synchronous CRCs were sporadic or Lynch-associated tumors. Furthermore, there were no data for palliative therapy for synchronous CRC patients with distant recurrence. Genetic information for *BRAF* mutation in included patients was not available in solitary CRC patients (*n* = 169, 29.1%), but all genetic information was available in synchronous CRC patients.

In conclusion, this study showed that the oncologic outcomes might differ according to the mutation burden characterized by *RAS, BRAF*, and MSI between synchronous CRC and solitary CRC. Furthermore, *RAS* and *BRAF* mutations were associated with worse DFS compared with MSI status, independently of clinical factors, stage, and tumor burden. Our systematic review highlighted a lack of data and much heterogeneity in the genetic characteristics and survival outcomes of synchronous CRC relative to that of solitary CRC. These factors make it difficult to predict the prognosis of synchronous CRC and complicate the decision-making process when selecting the most appropriate target drug following relapse of synchronous CRC.

## Data Availability

The datasets used and/or analyzed during the current study will be available from the corresponding author on reasonable request.

## Abbreviations

CRC: Colorectal cancer
MSI: Microsatellite instability
MMR: Mismatch repair
BRAF: V-Raf murine sarcoma viral oncogene homolog B1
HNPCC: Hereditary non-polyposis colorectal cancer
DFS: Disease-free survival
OS: Overall survival
